# Preoperative Iron Deficiency Is Associated With Increased Blood Transfusion in Infants Undergoing Cardiac Surgery

**DOI:** 10.3389/fcvm.2022.887535

**Published:** 2022-06-02

**Authors:** Peng Gao, Xu Wang, Peiyao Zhang, Yu Jin, Liting Bai, Wenting Wang, Yixuan Li, Jinping Liu

**Affiliations:** Pediatric Cardiac Surgery Center, Fuwai Hospital, National Center for Cardiovascular Diseases, Chinese Academy of Medical Sciences and Peking Union Medical College, Beijing, China

**Keywords:** iron deficiency, cardiac surgery, infants, blood transfusion, congenital heart disease

## Abstract

**Background:**

Iron deficiency (ID) is common in patients undergoing cardiac surgery, which is associated with adverse outcomes. However, the relevance of ID in congenital heart disease is still unclear. This study aimed to investigate the characteristics of preoperative ID and its association with clinical outcomes in infants undergoing cardiac surgery with cardiopulmonary bypass.

**Methods:**

In this retrospective study, 314 patients undergoing cardiac surgery were assigned into three groups according to their preoperative ID status. Absolute ID was defined by serum ferritin <12 μg/L, and functional ID was defined by serum ferritin level at 12–30 μg/L and transferrin saturation <20%. Baseline characteristics were compared between groups and multiple logistic regression was used to identify predictors for ID. The association between ID and clinical outcomes, including allogenic blood transfusion requirements, was also evaluated.

**Results:**

Among the 314 patients included, 32.5% were absolute ID and 28.7% were functional ID. Patients with absolute ID were more often of higher weight, cyanotic heart disease, and anemia. The presence of absolute ID was associated with an increase in postoperative blood transfusion (OR 1.837, 95% CI 1.016–3.321, *p* = 0.044). There was no significant difference in postoperative morbidity, mortality, and the length of hospital stay.

**Conclusions:**

Absolute ID was associated with preoperative anemia and cyanotic heart disease, and was an independent risk factor for postoperative blood transfusion. Further research should better explore the definition of ID and its impact on outcomes in pediatric cardiac surgery.

## Introduction

Iron deficiency (ID) is one of the main causes of anemia worldwide and is the most common micronutrient deficiency in children (1, 2). It is associated with adverse outcomes, affecting up to 50% of children in developing countries (1, 3). During early childhood, rapid growth and development exhaust iron stores, leading to increased exposure to ID (3). According to iron stores, ID can be divided into absolute iron deficiency (AID) and functional iron deficiency (FID) (4). In adults, AID is defined in presence of serum ferritin (SF) level <100 μg/L, and FID was defined by SF >100 μg/L combined with transferrin saturation (TAST) <20% (4, 5). Whereas children have different cutoff values depending on age and pathological conditions (1, 6).

Iron deficiency is frequently observed in adult cardiac surgery, which is recognized as a risk factor for increased red blood cell (RBC) transfusion, postoperative fatigue, and mortality (7–9). Currently, the prevalence of ID and its impact on clinical outcomes have not been as comprehensively studied in children, especially in infants. The aims of this study were to investigate the frequency and predictors of preoperative ID in infants undergoing cardiac surgery and to describe the association between ID and clinical outcomes.

## Materials and Methods

### Study Design and Patients

This was a single-center, retrospective cohort study conducted in Fuwai hospital (Beijing, China). From January 2020 to December 2021, every infant (1–12 months) who underwent initial cardiac surgery with cardiopulmonary bypass (CPB) in our institution qualified for the study. The exclusion criteria included patients with undetermined iron status and aged <6 months. Because there are rapid changes in storage iron concentrations in the first 6 months of life (6), and no appropriate definition of anemia is suitable for this population. Meanwhile, as an acute-phase reaction protein and inflammatory biomarker, high-sensitivity C-reactive protein (HSCRP) >3 mg/L was associated with a high risk of cardiovascular events and systemic inflammatory (10). In order to partially avoid the influence of inflammation on the diagnosis of ID, patients with HSCRP >3 mg/L were also excluded.

The study included 314 consecutive patients transferred to the pediatric intensive care unit (PICU) after cardiac surgery. As there was no comparable report available to develop a satisfactory hypothesis, a formal sample size calculation was not conducted. The sample size of 314 patients analyzed represented the largest cohort of infants (11, 12) and was comparable to the ID study in adults (5).

### Definitions

According to the WHO guideline (13), anemia was defined as hemoglobin (Hb) concentration <110 g/L for children 6–59 months of age. Thus, the definition of anemia in our study was Hb <110 g/L, regardless of the type of congenital heart disease. In previous studies on adult cardiac surgery, SF <100 μg/L was defined as AID, and SF >100 μg/L and TAST <20% was defined as FID (5). In children <5 years of age, the WHO (6) recommends that a SF concentration <12 μg/L indicates depleted iron stores, while SF <30 μg/L indicates ID during an acute phase response or chronic disease. Combining the above, AID was defined by SF <12 μg/L, and FID was defined by SF level at 12–30 μg/L and TAST <20% in the present study. The definition of cyanotic heart disease was based on the anatomy of congenital heart disease, including Tetralogy of Fallot (TOF), Double outlet right ventricle (DORV), Pulmonary stenosis (PS), Pulmonary atresia (PA), and Total anomalous pulmonary venous connection (TAPVC).

### Data Collection

On the day before the operation, the blood sample was systematically collected for blood biochemical examination, including iron status. The following variables were collected and analyzed. Preoperative data: demographics and laboratory assessments including Hb (g/L), serum bilirubin (μmol/L); serum creatinine (μmol/L); blood urea nitrogen (BUN) (mmol/L); HSCRP (mg/L), SF (μg/L) and TAST (%). Intra-operative data: type of surgery; CPB duration; intraoperative RBC consumption (RBC priming and transfusion). Postoperative data: requirement for allogeneic RBC transfusion; length of ICU stay, duration of mechanical ventilation, length of hospitalization, and acute kidney injury (an increase of creatinine >50% vs. preoperative value); inpatient mortality and morbidity. The postoperative morbidity was defined as pulmonary or incision infection, need for peritoneal dialysis, re-intubation or re-thoracotomy, and the requirement of extracorporeal membrane oxygenation. Data were collected through the computerized patient record system.

### Clinical Practice

Preoperative nutrition of all patients continued the daily recipes, with no special interventions, including iron supplementation, or dietary restrictions.

The procedure of cardiac surgery followed the standard protocol of the Pediatric Cardiac Surgery Center of Fuwai Hospital and was performed under general anesthesia and CPB. Every patient received a central venous catheter placed in the superior vena cava and a radial or femoral artery puncture. The CPB circuit consisted of a roller pump (Stockert S5, Sorin) and a hollow-fiber membrane oxygenator (Fx05, Terumo). Allogeneic RBC was added to the priming solution if the body weight was <8 kg or preoperative Hb was lower than 120 g/L. All patients underwent conventional ultrafiltration during CPB and modified ultrafiltration after CPB weaning.

Before aortic cannulation, 400 IU/kg heparin was administered to achieve an activated clotting time longer than 480 s, and a protamine dose of 4 mg/kg was administered for the reversal of the heparin effect after weaning of CPB. After surgery, all patients were admitted to PICU, which specialized in congenital heart disease, and received postoperative treatment by a fixed pediatric care team.

The decision to perform an intraoperative RBC transfusion was made if Hb <70 g/L during CPB, and Hb <90 g/L after CPB weaning. Whilst after surgery, the threshold for RBC transfusion was set at 80 or 90 g/L in the presence of signs suggestive of end-organ ischemia and low cardiac output.

### Statistical Analysis

To assess baseline characteristics and clinical outcomes, patients were assigned into three groups according to the definition criteria of ID as described above. The normal distribution of continuous variables was assessed visually using histograms and Q–Q plots. The normally distributed data were presented as mean with standard deviation, and non-normally distributed variables were presented as median with inter-quartile range. Differences among groups were compared using the Analysis of Variance or the Kruskal–Wallis test, depending on the normal distribution. Categorical variables were presented as frequency with percentage, and were analyzed with the χ^2^ test or Fisher exact test. As a *post hoc* analysis, Bonferroni's multiple comparison test was performed. To further elucidate the distribution of ID, patients were also grouped according to anemia or cyanotic heart disease, comparing differences in the distribution of ID and indexes of iron store among groups.

Multiple logistic regression was applied for the association between variables with AID and FID. Age, gender, and all variables with a *p* < 0.05 in Table 1 were added into a stepwise variable selection, reporting odds ratio (OR) with 95% confidence interval (CI) for the independent predictors identified. A multivariate logistic regression model was performed on RBC transfusion to determine AID as an independent risk factor. Possible confounders were chosen for clinical reasons, including age, gender, weight, cyanotic heart disease, preoperative Hb, CPB duration, and intraoperative RBC consumption. Moreover, the association of SF and postoperative RBC transfusion was evaluated by the restricted cubic spline model with four knots at the 5th, 35th, 65th, and 95th percentiles of SF.

**Table 1:** Baseline characteristics of the patient population.

	**Non-ID**	**Absolute ID**	**Functional ID**	***p*-Value**
	***n* = 122**	***n* = 102**	***n* = 90**	
Age (month)	8.14 (1.91)	8.65 (1.84)	8.24 (1.80)	0.109
Female sex	64 (52.5%)	42 (41.2%)	46 (51.1%)	0.202
Weight (kg)	7.25 (1.35)	7.84 (1.24)	7.32 (1.35)	0.002
Premature	14 (11.6%)	6 (5.9%)	9 (10.0%)	0.331
Cyanotic heart disease	21 (17.2%)	45 (44.1%)	11 (12.2%)	<0.001
Hemoglobin (g/L)	118.85 (16.14)	111.84 (14.87)	115.10 (13.26)	0.002
Preoperative anemia	29 (23.8%)	46 (45.1%)	29 (32.2%)	0.003
Platelet count (10^9^/L)	329 (114)	360 (109)	324 (87)	0.027
Albumin (g/L)	41.92 (3.34)	42.0 (2.04)	41.96 (4.02)	0.981
ALT (IU/L)	26 (19.75–41.25)	22 (16–31)	25 (18–36)	0.015
AST (IU/L)	50 (43.75–62)	51.5 (44–63.25)	52.5 (45.5–67)	0.382
Total bilirubin (μmol/L)	4.95 (3.51–7.12)	6.32 (4.49–8.88)	5.28 (3.88–7.15)	0.003
Direct bilirubin (μmol/L)	1.12 (0.78–1.72)	1.47 (1.16–2.06)	1.38 (0.96–1.68)	<0.001
Creatinine (μmol/L)	25.92 (6.99)	24.70 (6.43)	24.31 (5.59)	0.157
Blood urea nitrogen (mmol/L)	3.78 (1.65)	2.52 (1.03)	3.08 (1.23)	<0.001
Cystatin C (mg/L)	1.07 (0.21)	1.03 (0.14)	1.02 (0.16)	0.081
CK-MB (IU/L)	29.16 (18.01)	27.66 (15.80)	30.16 (21.67)	0.639
Lactic dehydrogenase (IU/L)	324.83 (87.94)	333.52 (87.43)	317.12 (98.62)	0.459
HSCRP (mg/L)	0.27 (0.16–0.56)	0.25 (0.11–0.49)	0.26 (0.15–0.41)	0.339
Serum iron (μmol/L)	10.85 (4.60)	5.91 (2.90)	7.87 (2.33)	<0.001
TIBC (μmol/L)	54.03 (7.98)	75.13 (15.08)	60.43 (7.82)	<0.001
Transferrin saturation (%)	20.11 (9.06)	8.43 (4.68)	13.17 (3.80)	<0.001
Serum ferritin (μg/L)	40.39 (31.94–55.42)	5.94 (4.15–9.0)	18.81 (15.22–24.62)	<0.001
Transferrin (g/L)	2.53 (0.35)	3.47 (0.67)	2.76 (0.38)	<0.001

*ID, iron deficiency; ALT, alanine aminotransferase; AST, aspartate aminotransferase; CK-MB, creatine kinase isoenzymes; HSCRP, hypersensitive C reactive protein; TIBC, total iron binding capacity*.

All analyses were carried out in SPSS 25.0 (IBM Corp., Armonk, NY, USA) and R 4.1.0 (The R Foundation for Statistical Computing, Vienna, Austria). A *p*-value of <0.05 was considered significant for all statistical tests.

## Results

### Study Population

**Figure 1:**
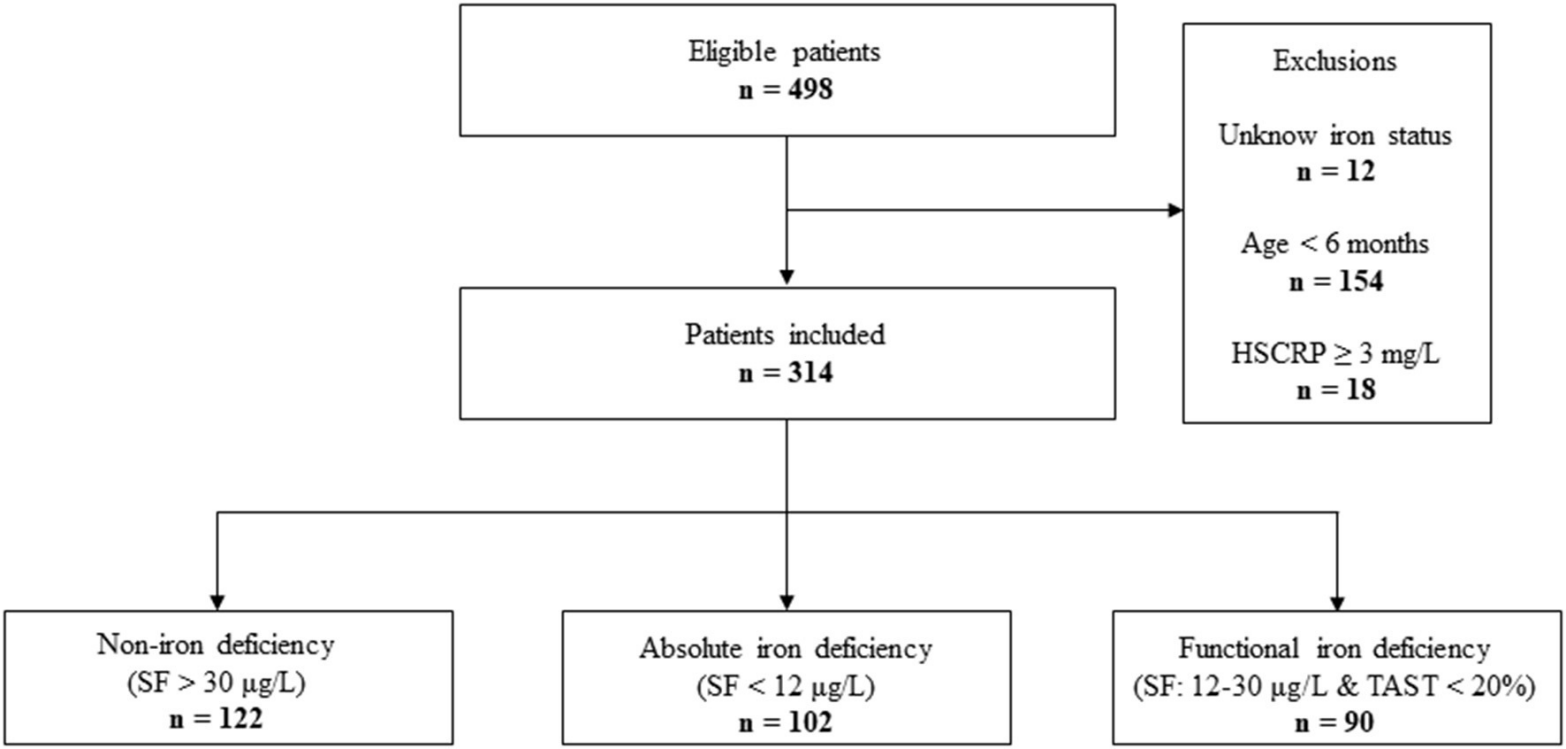
STROBE-style study flow chart outlining recruitment to and exclusion from the analysis sample. HSCRP, hypersensitive C reactive protein; SF, serum ferritin; TAST, transferrin saturation.

From January 2020 to December 2021, the data of 498 infants undergoing cardiac surgery in our institution was collected. A total of 184 patients were excluded from the final analysis. The reasons for exclusion were shown in Figure 1. The median age and weight of included patients (*n* = 314) were 8.33 ± 1.86 months and 7.46 ± 1.34 kg, respectively. The types of congenital heart disease were as follows: Ventricular septal defect 190 (60.5%), Atrial septal defect 35 (11.2%), TOF 55 (17.5%), DORV 9 (2.9%), Coarctation of the aorta 8 (2.6%), PS 7 (2.2%), Total endocardial cushion defect 5 (1.6%), PA 4 (1.3%), and TAPVC 2 (0.6%). All patients underwent CPB with a median duration of 78 (60–102.5) min (Supplementary Table 1).

### Characteristics and Predictors of ID

**Figure 2:**
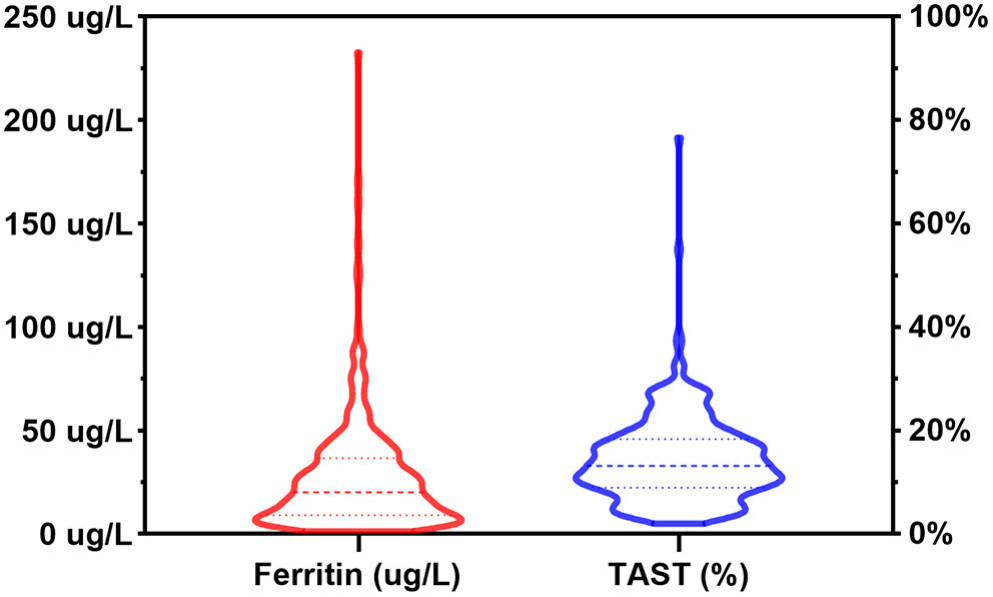
Distribution of serum ferritin levels and transferrin saturation in the patient population. TAST, transferrin saturation.

Figure 2 depicted the distribution of SF and TAST before surgery. According to the preoperative status of iron stores, ID was identified in 192 (61.2%) out of the 314 patients. The pattern of FID was found in 90 (28.7%) patients, and 102 (32.5%) patients were identified as AID. The baseline characteristics of patients were reported in Table 1. The group differences showed that patients with AID were more often of higher weight, cyanotic heart disease, and anemia (Hb <110 g/L). Furthermore, the Hb value was significantly lower in patients with AID vs. patients without ID or with FID (*p* = 0.002). However, there was no significant difference between the FID and non-ID patients. BUN and serum iron concentration was significantly lower in both FID and AID groups with respect to non-ID patients (*p* < 0.001). As a consequence of the definition of ID, SF levels and TAST were lower in patients with AID or FID vs. patients without ID (*p* < 0.001).

**Figure 3:**
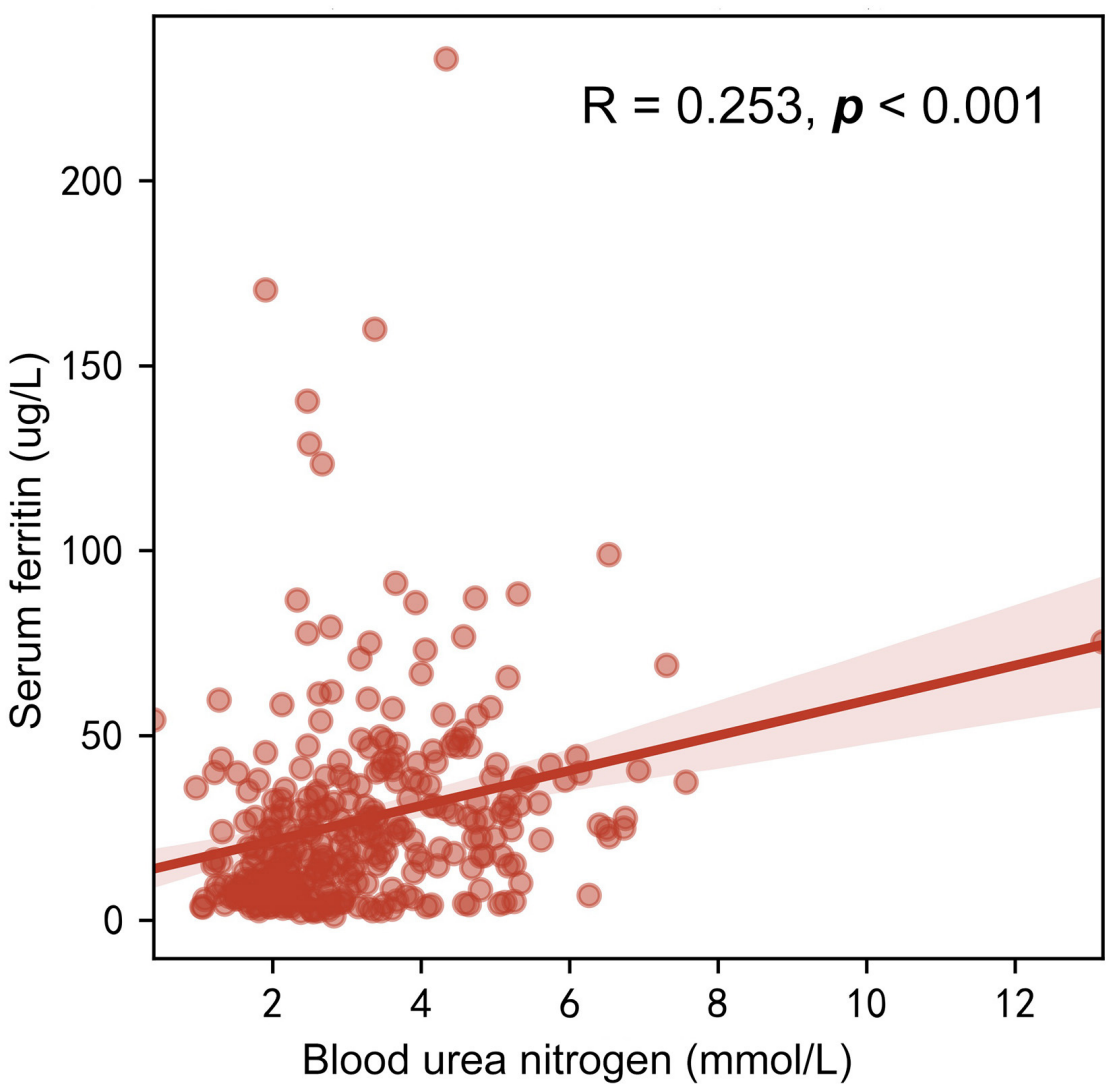
Correlation between serum ferritin and blood urea nitrogen.

Multiple logistic regression analysis for identification of the independent risk factors for ID was performed, variables including age, gender, and all factors with *p*-value < 0.05 at the univariate analysis in Table 1. Table 2 reported the results of this analysis: the independent predictors for AID were age, cyanotic heart disease, Hb, and BUN (*p* < 0.001). BUN was also significantly associated with FID (*p* = 0.001). Interestingly, BUN seemed to exert a “protective” effect for ID, which was correlated with SF (*R* = 0.253, *p* < 0.001; Figure 3), and the receiver operating characteristic (ROC) curve for BUN predicting the presence of ID had an AUC of 0.708 (95%CI 0.649–0.768, *p* < 0.001). Additionally, when taking anemia into the logistic regression instead of Hb, it was an independent risk factor for AID (OR 4.144, 95% CI 2.002–8.578, *p* < 0.001).

**Table 2:** Multiple logistic regression for iron deficiency.

	**Absolute iron deficiency**		**Functional iron deficiency**	
	**OR (95% CI)**	***p*-Value**	**OR (95% CI)**	***p*-Value**
Age (month)	1.418 (1.186–1.695)	<0.001	1.115 (0.950–1.309)	0.182
Cyanotic heart disease	8.892 (3.777–20.934)	<0.001	0.741 (0.296–1.863)	0.526
Hemoglobin (g/L)	0.939 (0.914–0.964)	<0.001	0.988 (0.966–1.011)	0.307
Blood urea nitrogen (mmol/L)	0.475 (0.357–0.632)	<0.001	0.689 (0.550–0.863)	0.001

*OR, odds ratio; CI, confidence interval*.

**Figure 4:**
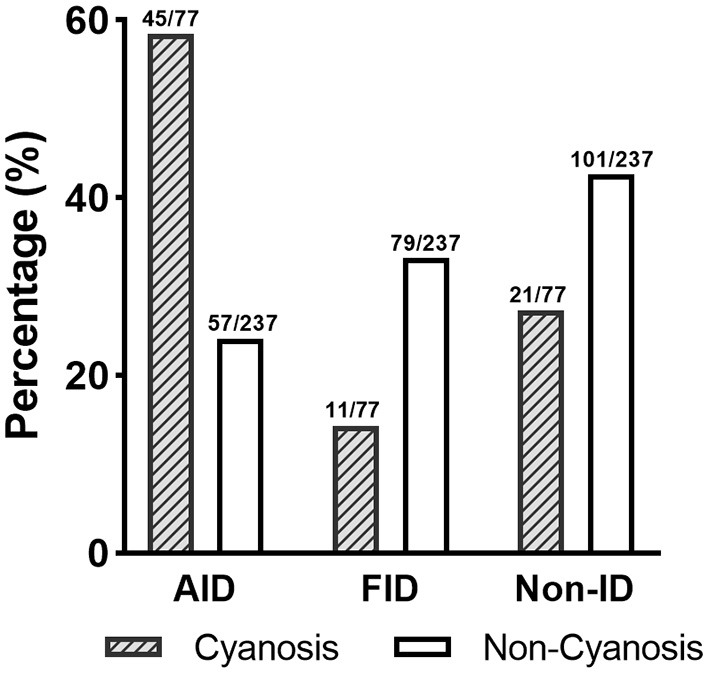
Distribution of ID patterns in patients with cyanotic heart disease. AID, absolute iron deficiency; FID, functional iron deficiency; non-ID, non-iron deficiency.

Before surgery, anemia (Hb <110 g/L) was found in 104 (33.1%) of the patient population. Within the patients with preoperative anemia, AID (44.2%) was observed more frequently than FID (27.9%) or non-ID (27.9%; Table 3). In contrast, most patients in non-anemia group presented with non-ID (44.3%; *p* = 0.003). Patients with anemia had significantly lower values of SF and TAST (*p* < 0.001), as well as lower levels of serum iron (*p* = 0.036). After adjusting for age, gender, weight, and cyanotic heart disease, AID was an independent risk factor for anemia (*p* < 0.001). In addition, patients with cyanotic heart disease were more likely to experience AID (*p* < 0.001; Figure 4), and with significantly lower levels of SF (*p* < 0.001) and TAST (*p* = 0.032; Table 4).

**Table 3:** Preoperative status of iron deficiency in anemia and non- anemia patients.

	**Anemia** ***n* = 104**	**Non-anemia** ***n* = 210**	***p*-Value**
Hemoglobin (g/L)	100.80 (7.12)	122.78 (12.64)	<0.001
Non-ID	29 (27.9%)	93 (44.3%)	0.005
Absolute ID	46 (44.2%)	56 (26.7%)	0.002
Functional ID	29 (27.9%)	61 (29.0%)	0.830
Serum ferritin (μg/L)	15.21 (7.02–31.67)	23.0 (10.0–37.73)	0.036
Transferrin saturation (%)	11.19 (6.41)	15.88 (8.59)	<0.001
Serum iron (μmol/L)	6.57 (3.04)	9.30 (4.28)	<0.001
TIBC (μmol/L)	64.48 (16.48)	61.85 (12.57)	0.117
Transferrin (g/L)	2.94 (0.70)	2.88 (0.60)	0.434

*ID, iron deficiency; TIBC, total iron binding capacity*.

**Table 4:** Preoperative status of iron deficiency in cyanosis heart disease patients.

	**Cyanosis heart disease** ***n* = 77**	**Non-cyanosis heart disease** ***n* = 237**	***p*-Value**
Non-ID	21 (27.3%)	101 (42.6%)	0.016
Absolute ID	45 (58.4%)	57 (24.1%)	<0.001
Functional ID	11 (14.3%)	79 (33.3%)	0.001
Hemoglobin (g/L)	125.09 (19.01)	112.38 (12.24)	<0.001
Preoperative anemia	12 (15.6%)	92 (38.8%)	<0.001
High Hb (≥140 g/L)	11 (14.3%)	2 (0.8%)	<0.001
Serum iron (μmol/L)	8.01 (5.17)	8.52 (3.71)	0.344
TIBC (μmol/L)	70.28 (13.79)	60.26 (13.21)	<0.001
Transferrin saturation (%)	12.58 (9.54)	14.89 (7.69)	0.032
Serum ferritin (μg/L)	9.37 (4.52–26.49)	23.18 (12.38–38.37)	<0.001
Transferrin (g/L)	3.32 (0.70)	2.77 (0.55)	<0.001

*ID, iron deficiency; TIBC, total iron binding capacity*.

### Effect of ID on Clinical Outcomes

As shown in Table 5, there was no statistic difference in postoperative duration of mechanical ventilation between groups, although it seemed longer in patients with AID, compared with FID and non-ID (*p* = 0.178). The length of stay in ICU and hospital, mortality, morbidity, and AKI were not different among groups. Patients with AID were more likely to receive a postoperative RBC transfusion (65.7%) compared with FID group (43.3%) and non-ID group (48.4%; *p* = 0.004).

**Table 5:** Clinical outcomes of the patient population.

	**Non-ID** ***n* = 122**	**Absolute ID** ***n* = 102**	**Functional ID** ***n* = 90**	***p*-Value**
MV (h)
Mean (SD)	34.43 (78.92)	48.21 (212.87)	33.2 (118.76)	0.178
Median (IQR)	9 (5–23.25)	11 (6–22)	8 (5–15.75)	
ICU stay (day)
Mean (SD)	3.87 (5.38)	4.68 (10.63)	3.74 (6.91)	0.696
Median (IQR)	2 (1–4)	2 (1–4)	2 (1–4)	
Hospital stay (day)
Mean (SD)	14.78 (9.15)	15.48 (11.02)	14.22 (9.88)	0.652
Median (IQR)	13 (9.75–16)	13 (9–17)	12.5 (9–16)	
ICU transfusion	59 (48.4%)	67 (65.7%)	39 (43.3%)	0.004
Mortality	0	1 (1.0%)	0	0.353
Morbidity	17 (13.9%)	12 (11.8%)	11 (12.2%)	0.876
AKI	50 (41.0%)	42 (41.2%)	33 (36.7%)	0.677

*ID, iron deficiency; MV, mechanical ventilation; ICU, intensive care unit; AKI, acute kidney injury*.

**Figure 5:**
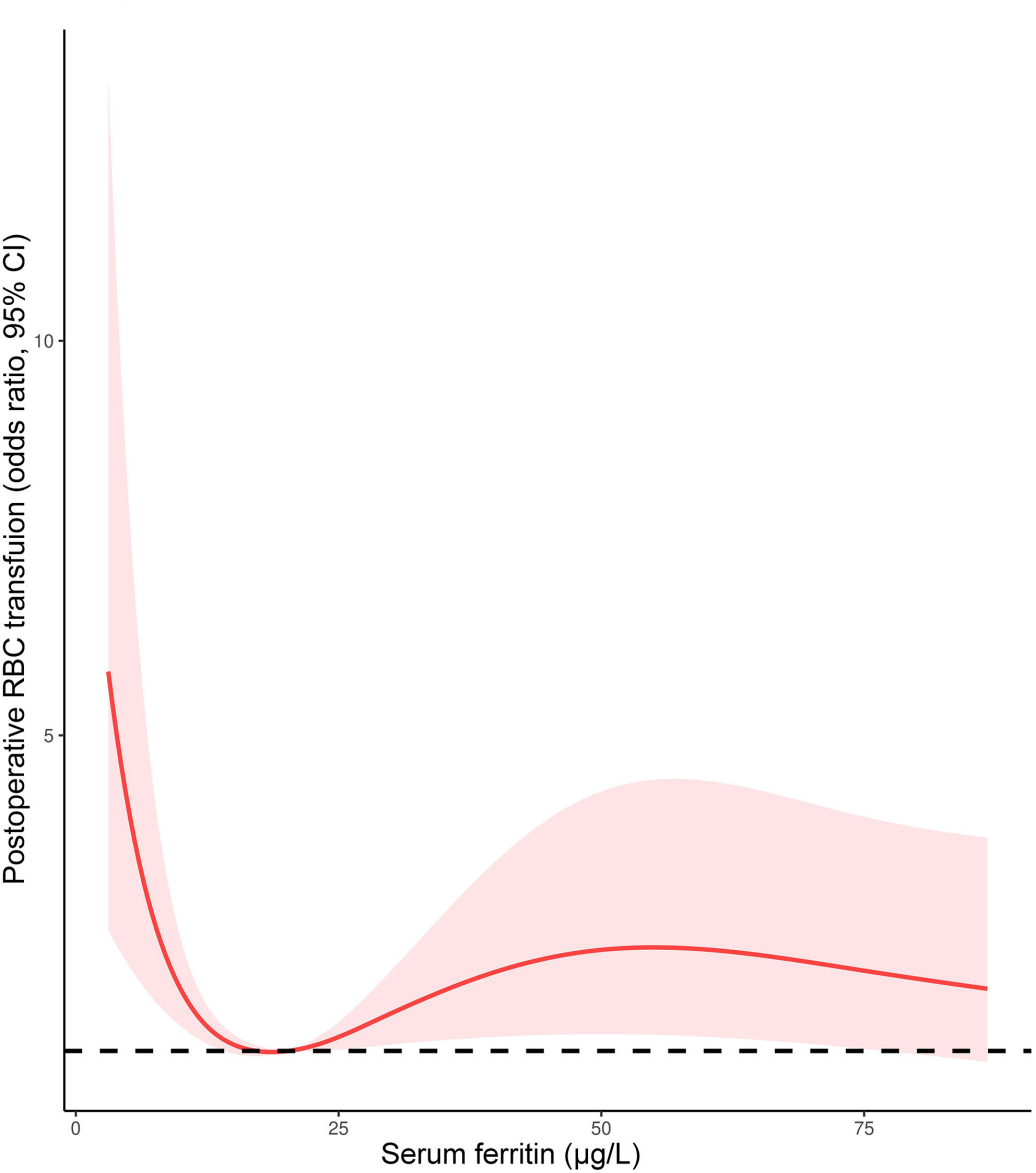
The restricted cubic spline model for serum ferritin and postoperative RBC transfusion.

Given the proportion of postoperative RBC transfusion was comparable in patients with FID and non-ID, a multivariate logistic regression model was performed to determine the association of AID with RBC transfusion. After adjusting for age, gender, weight, cyanotic heart disease, preoperative Hb, CPB duration, and intraoperative RBC consumption, AID was an independent risk factor for postoperative RBC transfusion (OR 1.837, 95% CI 1.016–3.321, *p* = 0.044; Table 6). Additionally, the restricted cubic spline model confirmed a nonlinear association between SF and RBC transfusion (*p*-Nonlinear = 0.001), with a cutoff value of 20.225 (Figure 5). The ROC curve for SF also showed a prediction power for RBC transfusion (*p* = 0.035), with an AUC of 0.431 (95% CI 0.368–0.495).

**Table 6:** Multivariate logistic regression for postoperative RBC transfusion.

	**OR (95% CI)**	***p*-Value**
Gender (Male)	1.697 (1.010–2.854)	0.046
Age (month)	0.950 (0.821–1.100)	0.493
Weight (kg)	0.754 (0.603–0.943)	0.013
Cyanotic heart disease	1.727 (0.845–3.530)	0.134
Hemoglobin (g/L)	0.989 (0.970–1.007)	0.233
CPB Duration (min)	1.018 (1.010–1.027)	<0.001
Intraoperative RBC consumption (U)	0.329 (0.141–0.764)	0.010
Absolute iron deficiency	1.837 (1.016–3.321)	0.044

*OR, odds ratio; CI, confidence interval; CPB, cardiopulmonary bypass; RBC, red blood cell*.

## Discussion

In this study, we demonstrated that AID (SF <12 μg/L), independent of anemia and cyanosis heart disease, is a risk factor for the requirements of RBC transfusion after infant cardiac surgery. As far as we know, this is the first study showing the characteristics and the effect of preoperative ID in infants undergoing cardiac surgery. The frequency of postoperative RBC transfusion was significantly higher for patients with AID than those with FID and without ID. After adjustment for confounders, a preoperative serum ferritin <12 μg/L was associated with 1.8 times increased risk of postoperative RBC transfusion. We chose the confounders based on a review (14) and clinical reasons, including age, gender, weight, cyanotic heart condition, preoperative Hb, and intraoperative RBC consumption. As there were many different types of congenital heart surgery, we used cyanotic heart condition for classification. In addition, as the duration of CPB could partially represent the complexity of the procedure, it was added as a confounder in the logistic regression model.

Iron deficiency is a prevalent malnutrition that affects up to two billion people globally, especially infants (1). Apart from erythropoiesis, iron has integral roles in several organ systems, including oxygen transport and storage, mitochondrial activity, cell signaling, and gene expression (15). Most of the circulating iron is consumed by erythropoiesis. Muscle growth and production of new myoglobin are also important consumers of iron in children (3). Since infants have high iron requirements due to rapid growth and development, they may potentially be at increased risk of ID if not supplemented with adequate iron-rich formulas or supplements (3). Our results showed that AID patients were of higher weight than FID or non-ID, which might also reflect this. The distribution of ID in children undergoing cardiac surgery is not well characterized, particularly in cyanotic children in whom ID can exist even with increased Hb levels (14). This was also shown in our data, where patients with cyanotic heart disease had higher preoperative Hb levels and less anemia, but most of them (58.4%) had AID (Table 4). A possible explanation is that abnormal erythropoiesis in patients with cyanotic heart disease may expose them to more severe iron depletion and consequently lead to an increased risk of AID.

In adults, the definition of ID included two different patterns, AID and FID, defined by SF <100 mg/L and SF <300 mg/L combined with TAST <20%, respectively (4, 5). Whilst in current WHO guideline (6), a cut-off value of 12 mg/L was recommended to define ID in children younger than 5 years old, and 30 mg/L in the presence of inflammation. Consequently, our definition of ID combined the definition of ID in adult cardiac surgery with the characteristics of iron metabolism in children, defining AID as SF <12 mg/L and FID as SF at 12–30 mg/L and TAST <20%. In previous studies in children, ID was defined as the presence of ≥2 of the following 4 criteria: serum iron <50 mg/dl, SF <20 ng/ml, transferrin >300 ng/ml, and TAST <15% (11, 12). However, this definition failed to distinguish between AID and FID, two different patterns of ID that have been generally demonstrated in adults (5, 16). To further clarify the effect of ID, it would be valuable to examine the difference of AID and FID in the associated risk of morbidity, RBC transfusion and response to iron treatment (16).

Our results were in line with previous studies in adult cardiac surgery, which showed ID to be associated with increased transfusions of allogeneic blood products (7, 9). Rossler et al. (8) indicated that postoperative transfusions of allogenic blood products within 90 days were considerably increased in ID (SF <100 mg/L) patients with or without anemia. The effect of ID on RBC transfusion was shown to have an OR of 1.8 (95% CI: 1.2–2.6) in multivariate regression analysis (8), which was similar to our findings. Additionally, even with a higher prevalence of preoperative anemia, patients with FID had a comparable rate of postoperative RBC transfusion with the non-ID group. Furthermore, restricted cubic spline plots showed that the level of SF at the lowest risk for postoperative RBC transfusion is 20 mg/L, which is within the definition of FID. Perhaps a better definition of ID was needed, for example with hepcidin and soluble transferrin receptors, which showed advantages for the diagnosis of ID (6).

In general, the postoperative course of patients with FID was not different from that of non-ID patients (5). Contrary to FID, AID was associated with a worse postoperative course, including increased mortality and prolonged hospital stay (5, 8). Contrary to expectations, we did not find a significant difference in clinical outcomes such as duration of mechanical ventilation and length of ICU stay between groups. Miles et al. (17) also reported the weak association between ID and outcomes. The impact of different patterns of ID on clinical outcomes remains to be further explored.

Patients with heart failure and ID showed symptomatic improvements from intravenous iron administration, irrespective of the presence of anemia (15). Whilst all patients might benefit from the treatment of preoperative ID (4), patients undergoing cardiac surgery could especially do so (18). This might be attributable to the physiological role of iron for the heart, and its essential role for mitochondrial function and cellular bioenergetics (19). Spahn et al. reported that an ultrashort-term treatment of isolated ID resulted in a higher postoperative Hb and reduced transfusions of allogeneic blood products in anemia patients with no significant negative side-effects (18). In addition, oral iron supplementation increased preoperative Hb levels in children undergoing cardiac surgery (20). As allogenic transfusions are associated with an increase of adverse events (21, 22), children undergoing cardiac surgery may benefit from a reduced requirement for postoperative RBC transfusion.

Nevertheless, a retrospective study showed supplementation with intravenous iron did not improve outcomes in patients with iron deficiency anemia and the transfusion requirement was significantly higher than the non-anemic cohort (23). Myles et al. (24) even believed that intravenous iron therapy should not be recommended since it was costly and offered a modest effect on the reduction in transfusion requirements. When the diagnosis of iron deficiency anemia is made preoperatively, treatment with oral iron supplements may be inadequate to achieve timely correction in children (14). However, as the iron regulatory hormone, hepcidin increases in inflammatory state, regardless of the presence or absence of infection (25). It impairs the function of the ferroportin, preventing the transport of iron across basement membranes, and thereby influencing the role of ID therapy in preoperative anemia (16). Newer intravenous iron preparations, like ferumoxytol and ferric iron gluconate, offer safer means for rapid iron replacement (26). Currently, larger studies to clarify the effect of preoperative treatment of ID are warranted in children undergoing cardiac surgery. Furthermore, regardless of the efficacy of the treatment, ID should be considered an additional risk factor for cardiac surgery.

There were some limitations in our study. First, as a single-center study, the external validity of our results might be limited. Second, although we excluded patients with high baseline HSCRP in order to partially avoid the impact of the inflammatory on SF. Unfortunately, the lack of data on CRP or hepcidin made the diagnosis of ID less accurate. In addition, specific reasons for RBC transfusion, and the causes of anemia or coagulation/bleeding disorders were not analyzed in current study due to the limitation of data. The effect of ID on postoperative RBC transfusion remains to be further studied. Finally, the small sample might limit our statistical power to detect significant differences in clinical outcomes. Therefore, it was difficult to further compare the effects of different patterns of ID on outcomes in anemic and non-anemic patients. Future studies should include more patients and extend the current analysis by distinguishing between different types of congenital heart disease.

## Conclusion

Preoperative ID was a common comorbidity in infants undergoing cardiac surgery, including AID and FID. There was almost no difference between FID and non-ID patients. Whereas AID was an independent risk factor for postoperative RBC transfusion, with its own predictors including age, cyanosis heart disease, anemia, and BUN. Since patients might benefit from the treatment of ID and thus improved clinical outcomes, Further studies should better explore the definition of ID and its impact on outcomes in pediatric cardiac surgery.

## Data Availability Statement

The raw data supporting the conclusions of this article will be made available by the authors, without undue reservation.

## Ethics Statement

The studies involving human participants were reviewed and approved by Ethics Committee of Fuwai Hospital. Written informed consent from the participants' legal guardian/next of kin was not required to participate in this study in accordance with the national legislation and the institutional requirements.

## Author Contributions

PG, XW, PZ, YJ, LB, YL, WW, and JL contributed to conception and design of the study. PG, XW, and YJ organized the database. PG and PZ performed the statistical analysis. PG wrote the first draft of the manuscript. XW and JL revised the manuscript. All authors contributed to manuscript revision, read, and approved the submitted version.

## Funding

This study was supported by CAMS Innovation Fund for Medical Sciences (CIFMS) (Project Number: 2020-I2M-C&T-B-063).

## Conflict of Interest

The authors declare that the research was conducted in the absence of any commercial or financial relationships that could be construed as a potential conflict of interest.

## Publisher's Note

All claims expressed in this article are solely those of the authors and do not necessarily represent those of their affiliated organizations, or those of the publisher, the editors and the reviewers. Any product that may be evaluated in this article, or claim that may be made by its manufacturer, is not guaranteed or endorsed by the publisher.
