# Supplementary material for: Preoperative Iron Deficiency Is Associated With Increased Blood Transfusion in Infants Undergoing Cardiac Surgery
Source: Front Cardiovasc Med. 2022 Jun 2;9:887535. doi: 10.3389/fcvm.2022.887535 (PMC9200962; doi:10.3389/fcvm.2022.887535)
Supplement: Supplementary file 1 [file Table_1.docx]

Supplementary Material

**Supplementary Table 1.** Surgical characteristics of the patient population.

| **Type of congenital heart disease** |  |
| --- | --- |
| Ventricular septal defect | 190 (60.51%) |
| Atrial septal defect | 35 (11.15%) |
| Tetralogy of Fallot | 55 (17.52%) |
| Double outlet right ventricle | 9 (2.87%) |
| Coarctation of the aorta | 8 (2.55%) |
| Pulmonary stenosis | 7 (2.23%) |
| Total endocardial cushion defect | 5 (1.59%) |
| Pulmonary atresia | 4 (1.27%), |
| TAPVC | 2 (0.64%). |
| **Surgical characteristics** |  |
| chromosomal syndromes | 12 (3.82%) |
| CPB duration (min) | 78 (60-102.5) |
| ACC duration (min) | 50 (35-70) |
| Conventional ultrafiltration (mL) | 228.76±106.29 |
| Modified ultrafiltration (mL) | 114.87±27.59 |
| **RACHS** |  |
| 1 | 35 (11.14%) |
| 2 | 201 (64.01%) |
| 3 | 78 (24.84%) |
| **Intraoperative RBC consumption** |  |
| 0 U | 25 (7.96%) |
| 1 U | 281 (89.49%) |
| 2 U | 8 (2.55%) |

TAPVC, total anomalous pulmonary venous connection; CPB, cardiopulmonary bypass; ACC, aorta cross-clamping, RACHS, Risk Adjustment for Congenital Heart Surgery; RBC, red blood cell.
